# Oxytetracycline-resistant *Paenibacillus larvae* identified in commercial beekeeping operations in Saskatchewan using pooled honey sampling

**DOI:** 10.1177/10406387231200178

**Published:** 2023-09-13

**Authors:** Oleksii Obshta, Michael W. Zabrodski, Tayab Soomro, Geoff Wilson, Fatima Masood, Jenna Thebeau, Marina C. B. Silva, Sarah Biganski, Ivanna V. Kozii, Roman V. Koziy, M. Fahim Raza, Midhun S. Jose, Elemir Simko, Sarah C. Wood

**Affiliations:** Departments of Veterinary Pathology, University of Saskatchewan, SK, Canada; Prairie Diagnostic Services, Saskatoon, SK, Canada; Departments of Veterinary Pathology, University of Saskatchewan, SK, Canada; Crops and Irrigation Branch, Ministry of Agriculture, Government of Saskatchewan, Prince Albert, Saskatchewan, Canada; Veterinary Microbiology, University of Saskatchewan, SK, Canada; Departments of Veterinary Pathology, University of Saskatchewan, SK, Canada; Departments of Veterinary Pathology, University of Saskatchewan, SK, Canada; Departments of Veterinary Pathology, University of Saskatchewan, SK, Canada; Prairie Diagnostic Services, Saskatoon, SK, Canada; Departments of Veterinary Pathology, University of Saskatchewan, SK, Canada; Departments of Veterinary Pathology, University of Saskatchewan, SK, Canada; Departments of Veterinary Pathology, University of Saskatchewan, SK, Canada; Departments of Veterinary Pathology, University of Saskatchewan, SK, Canada; Departments of Veterinary Pathology, University of Saskatchewan, SK, Canada

**Keywords:** American foulbrood, antimicrobial resistance, honey bees, oxytetracycline, *pMA67*, *tet*(L)

## Abstract

American foulbrood (AFB) is an infectious disease of honey bee brood caused by the endospore-forming bacterium *Paenibacillus larvae. P. larvae* spores are resilient in the environment, thus colonies with clinical signs of AFB are often destroyed by burning to eradicate the causative agent. To prevent outbreaks of AFB, oxytetracycline metaphylaxis is widely used in North America, resulting in sustained selective pressure for oxytetracycline resistance in *P. larvae*. To determine if antimicrobial resistance (AMR) is present among *P. larvae* isolates from commercial beekeeping operations in Saskatchewan, Canada, we performed antimicrobial susceptibility testing of 718 *P. larvae* samples cultured from pooled, extracted honey collected from 52 beekeepers over a 2-y period, 2019 and 2020. We found that 65 of 718 (9%) *P. larvae* samples collected from 8 beekeepers were resistant to oxytetracycline with minimum inhibitory concentration (MIC) values of 64–256 µg/mL. Eight of 718 (1%) samples from 4 beekeepers had intermediate resistance to oxytetracycline (MIC: 4–8 µg/mL). Susceptibility testing for tylosin and lincomycin indicated that *P. larvae* in Saskatchewan continue to be susceptible to these antimicrobials (tylosin MIC: <1 µg/mL, lincomycin MIC: ≤2 µg/mL). Most oxytetracycline-resistant *P. larvae* samples were identified in northeastern Saskatchewan. Whole-genome sequence analysis identified the *P. larvae*–specific plasmid *pMA67* with tetracycline-resistance gene *tet*(L) in 9 of 11 oxytetracycline-resistant *P. larvae* isolates sequenced. Our results highlight the advantage of using pooled, extracted honey as a surveillance tool for monitoring AMR in *P. larvae*.

Within the global beekeeping industry, there are 2 contrasting strategies for the prevention of American foulbrood (AFB), which is a highly contagious, invariably fatal disease of honey bee larvae and pupae caused by the endospore-forming, gram-positive bacterium *Paenibacillus larvae*.^17,39,41^ The first, employed by the European Union, New Zealand, and other countries in which antibiotic use in beekeeping is prohibited, is a best management practices (BMP) approach, involving frequent inspection and replacement of brood frames, strict sanitation and biosecurity practices, and destruction by burning or irradiation of colonies with clinical AFB to eliminate the resilient endospores of *P. larvae*.^24,26^ The second AFB-control strategy, used by North American beekeepers for the past 70 y, is antimicrobial metaphylaxis with oxytetracycline hydrochloride (OTC), in addition to BMP practices. In our local area of Saskatchewan (SK), Canada, 39% and 42% of beekeepers reported using OTC in spring and fall, respectively, for metaphylaxis of AFB.^12^ Lincomycin hydrochloride (LMC) and tylosin tartrate (TYL) have also been approved for AFB metaphylaxis in Canada and the United States.^31,42^ The projected economic impact of an AFB outbreak in spring, assuming 17% of colonies are clinically affected and destroyed by burning, has been estimated at $656 CAD per colony, underscoring the importance of effective AFB control to maintain the profitability of commercial beekeeping operations, which often manage >1,000 colonies per operation.^1^

Alarmingly, since ~2000, the emergence of OTC resistance within *P. larvae* in North America,^4,36^ Italy,^4^ and Argentina,^3^ and, more recently, the identification of LMC- and TYL-resistant *P. larvae* in the United States,^4^ threaten continued reliance on antimicrobials for AFB control in North America, and argue for an alternative, evidence-based approach to antimicrobial use in beekeeping. Accordingly, to guide antibiotic treatment decisions for AFB management, our research team developed an AFB surveillance tool that established prognostic thresholds for apiary-level risk of AFB based on *P. larvae* spore load in pooled, extracted honey.^47,48^

Similarly, we hypothesized that pooled, extracted honey may be useful to determine the prevalence of phenotypic and genotypic determinants of antimicrobial resistance (AMR) in *P. larvae*, including plasmid-mediated *tet*(L) and *tet*(K) genes, known to encode membrane-associated tetracycline efflux pumps in *P. larvae.*^4,25,36^ Moreover, genomic analysis of *P. larvae* isolates in honey will support molecular epidemiologic investigation of *P. larvae* spread, with the aid of established enterobacterial repetitive intergenic consensus (ERIC) genotyping^7,23^ and multilocus sequence typing (MLST) schemes^35^ for *P. larvae*.

The prevalence of AMR within *P. larvae* in our local beekeeping industry in SK, Canada is unknown, although *P. larvae* spores have been detected in >50% of SK honey samples.^47,48^ Thus, to complement our previous AFB surveillance and risk assessment, and support judicious and evidence-based antimicrobial use in the beekeeping industry, our objectives were to 1) perform antimicrobial susceptibility testing of SK isolates of *P. larvae* cultured from pooled, extracted honey, and 2) evaluate, through whole-genome sequence (WGS) analysis, genetic determinants of AMR and phylogenetic relatedness of a subset of *P. larvae* isolates, representative of the range of AMR phenotypes determined in objective 1.

## Materials and methods

### *Paenibacillus larvae* samples, culture media, and antibiotics

*P. larvae* isolates were revived from 718 *P. larvae* samples collected in 2019 and 2020 and stored at −80°C in 20% glycerol. These samples included spore suspensions and *P. larvae* isolates derived from 718 pooled, extracted honey samples representing 52 commercial beekeeping operations that collectively manage 81,000 of 110,000 (74%) of registered colonies in SK. Briefly, for each enrolled commercial beekeeping operation, 3 samples of extracted honey were collected from each of 6, randomly selected, geographically distinct apiaries per year. Each extracted honey sample was collected from a separate extraction load to prevent overlap in sampling honey from the same frames and/or hives. Accordingly, the 3 extracted honey samples per apiary represented 9–18 hives within the apiary. Taken together, the honey samples from the 52 commercial beekeepers in our study over 2 y represented 5–10% of the 110,000 colonies registered in SK.^48^ Stored samples were cultured in MYPGP medium^18^ with the addition of 3 mM L-tyrosine and 3 mM uric acid to enhance the germination of *P. larvae* spores (hereafter enhanced MYPGP [eMYPGP] agar and broth).^5,47^ Stocks of OTC (Thermo Fisher), TYL (Acros Organics, Thermo Fisher), and LMC (Alfa Aesar, Thermo Fisher) were prepared as 3 mg/mL, 1.5 mg/mL, and 0.1875 mg/mL stock solutions, respectively, filter-sterilized using 0.22-µm filters, and stored at −20°C until use.

### Screening of 718 *P. larvae* samples for susceptibility to OTC, TYL, and LMC

To screen *P. larvae* samples for susceptibility to OTC, the samples were divided into 2 groups, based on previously determined spore concentrations^44^: 1) samples with >3 spores/g (284 samples); and 2) samples with <3 spores/g (434 samples; Suppl. Table 1). For samples with >3 spores/g, to preserve genetic heterogeneity, 100 µL of spore suspension (representing the spore load per g of the original pooled honey sample) was plated on control and selective eMYPGP agar plates containing 2 μg/mL OTC. For samples with <3 spores/g, to avoid the difficulty of re-culturing samples with low spore concentrations, a single vegetative bacterial clone saved from the original culture^48^ of each spore suspension (stored at −80°C in eMYPGP broth and 20% glycerol) was plated on control and eMYPGP agar plates containing 2 µg/mL OTC. Plates were incubated for 72 h at 37°C with 5% CO_2_,^17^ and colony counts were performed. The concentration of OTC reflects the Clinical and Laboratory Standards Institute (CLSI) susceptibility breakpoints for *P. larvae*.^15^ *P. larvae* isolates that did not grow on 2 µg/mL OTC eMYPGP plates were interpreted to have a minimum inhibitory concentration (MIC) of ≤2 µg/mL OTC.

A modified version of the above methodology for OTC susceptibility screening was performed to screen for TYL and LMC resistance (Suppl. Table 2). Briefly, to screen the 284 samples with >3 spores/g for susceptibility to TYL and LMC, 100 µL of each spore suspension was pooled into groups containing a maximum of 15 spore suspensions per group, representing 1 beekeeping operation. After pooling, 100 µL of each sample was plated on control and eMYPGP agar plates containing 1 µg/mL TYL or 0.125 µg/mL LMC. LMC susceptibility testing was only possible for 693 of 718 *P. larvae* samples because of an overgrowth of contaminant bacteria in the remaining samples. Plates were incubated for 72 h at 37°C with 5% CO_2_,^16^ and colony counts were performed. The concentration of TYL represents 1/8 of the CLSI MIC susceptible breakpoint for *P. larvae*.^15^ A low concentration of LMC was arbitrarily selected given a lack of CLSI MIC breakpoints for *P. larvae*.

### MIC and MBC determination for OTC and LMC

*P. larvae* samples exhibiting growth on the antibiotic-containing plates during the preliminary screening procedure described above were subcultured for determination of MIC and minimum bactericidal concentration (MBC). Briefly, for MIC determination, up to 3 cfu per *P. larvae* sample, representing up to 3 biological replicates, were each subcultured in 5 mL of eMYPGP broth for 24 h at 37°C with 5% CO_2_ and then adjusted with sterile PBS to an optical density of 1.0 McFarland unit (OD_600_ 0.25). A set of three, 10-µL aliquots from each subculture, representing 3 technical replicates, were each added to a testing well in a 96-well plate containing 2-fold dilutions of either OTC (256–2 µg/mL) or LMC (8–0.125 µg/mL), prepared in 100 µL of eMYPGP broth. A positive control containing bacterial inoculum without antibiotic and a negative control containing only eMYPGP broth were included for each technical replicate. Bacterial growth was evaluated after 48 h incubation at 37°C with 5% CO_2_, and the MIC was interpreted as the minimum antibiotic concentration in a tested well that prevented visible *P. larvae* growth. In the case of a discrepancy in the MIC for *P. larvae* among technical replicates, the MIC was interpreted as the highest MIC of antibiotic obtained from all replicates. For quality control, MIC determination for *Staphylococcus aureus* ATCC 29253 was performed using the aforementioned technique.^13,16^

For MBC determination, the MIC plate wells containing 2-fold and 4-fold greater antibiotic concentrations than the MIC were subcultured in 3 technical replicates on eMYPGP agar plates and incubated for 24 h at 37°C with 5% CO_2_. The MBC was interpreted as the antibiotic concentration that resulted in the absence of *P. larvae* growth on eMYPGP agar.

### Kirby–Bauer disk diffusion susceptibility test for OTC and LMC

A Kirby–Bauer disk diffusion susceptibility test^6^ was performed for OTC and LMC for all resistant *P. larvae* isolates based on MIC and MBC determination (73 isolates used for OTC disk diffusion testing [Suppl. Table 3], and 100 randomly selected biological replicates [representing 78 isolates with 1–3 biological replicates per isolate] that grew on 0.125 µg/mL LMC used for LMC disk diffusion testing). Disk diffusion testing was performed using disks containing 30 µg of OTC (BD) or 2, 10, 4, and 15 µg of LMC (Oxoid); 4-µg LMC disks were not commercially available and were prepared manually by inoculating sterile, 6-mm paper disks (BD) with 20 µL of 200 µg/mL LMC.

To prepare bacterial inoculum for Kirby–Bauer testing, 3 biological replicates of each *P. larvae* isolate tested were subcultured and prepared as a 1.0 McFarland unit suspension as described above. Each bacterial suspension was inoculated on eMYPGP agar plates using a sterile cotton swab dipped into the suspension and spread evenly over the plate. Antibiotic-containing disks (3 technical replicates per antibiotic concentration per plate) were applied with sterile forceps, plates were incubated for 48 h at 37°C, and the inhibition zones were measured in mm. For quality control, Kirby–Bauer disk diffusion susceptibility testing to OTC was performed for *S. aureus* ATCC 25923 using the aforementioned procedure.^16^

### WGS analysis of 17 *P. larvae* isolates

To investigate the relationship between phenotypic resistance to OTC and AMR genes (ARGs), WGS was performed on 17 *P. larvae* isolates collected from 11 commercial beekeeping operations in SK, Canada. The isolates represented a range of sensitivity to OTC, including 3 susceptible isolates, 3 isolates with intermediate resistance, and 11 resistant isolates (Suppl. Table 3). Selected isolates were grown on eMYPGP medium^5^ for 3 d, then harvested for DNA extraction. WGS of all 17 isolates was performed by Prairie Diagnostic Services (Saskatoon, SK, Canada) using a GridION sequencing device (ONT; Oxford Nanopore Technologies).

To verify WGS quality and repeatability, 4 *P. larvae* isolates (PL002–PL005) were re-sequenced using a paired-end, 250-bp sequencing platform (MiSeq; Illumina), performed by Genome Quebec. For MiSeq WGS, DNA was extracted (DNeasy blood and tissue extraction kit; Qiagen), and libraries were generated from 100 ng of DNA (NEBNext Ultra II DNA library prep kit for Illumina; New England BioLabs). For ONT WGS, DNA was extracted (MagAttract HMW DNA kit; Qiagen), and library preparation was performed using 1,500 ng of DNA (Ligation sequencing [SQK-LSK109], and Native barcoding expansion 96 [EXP-NBD196] kits; Oxford Nanopore Technologies). Assembly of the 250-bp, paired-end Illumina raw reads was performed (TadPole assembler in Geneious Prime 2022.0.1), and assembly of the ONT raw reads was performed (Flye v.2.9^30^; Suppl. Table 4). Genome assembly completeness was assessed (BUSCO; Suppl. Table 5).^44^ WGS of 17 representative isolates of *P. larvae* have been deposited in GenBank under BioProject PRJNA948666.

WGS data were screened for genetic determinants of AMR (ResFinder v.4.1^10,11,49^ and the Comprehensive Antimicrobial Resistance Database [CARD]).^2^ Criteria for positive identification of an ARG included a minimum of 80% identity and 80% coverage of sequence length. The *tet*(L) resistance plasmid *pMA67*,^36^ as well as unnamed *P. larvae* plasmids 1 and 2,^7^ was investigated by mapping the reference sequences of these plasmids (NC_010875, NZ_CP019660, NZ_CP019661, respectively) to the query genome using BLAST.^11^ The BLAST search was performed with an *E*-value cutoff of 1 × 10^-5^, and a plasmid was considered to be present if the mapped sequences covered ≥80% of the reference length with ≥80% of sequence identity. All of the reference sequences were obtained from GenBank.

The multilocus sequence type (ST) of each isolate was determined by comparing the isolate’s genome against the *P. larvae* PubMLST database (https://pubmlst.org/organisms/paenibacillus-larvae)^27,35^ to assign an allele number for each of the 7 reference genes that comprise the MLST scheme for *P. larvae*. For 13 of 17 isolates sequenced with ONT (PL003, PL006–PL018; Suppl. Table 6), the *Natrans* gene in the MLST scheme had a 1-bp deletion, present in both the raw and assembled reads within a repetitive sequence of adenosine nucleotides, which are known to be vulnerable to technical error when sequencing using ONT.^14,33^ Re-sequencing of *P. larvae* isolates PL002–PL005, using the MiSeq platform, revealed that the 1-bp deletion was absent in the *Natrans* gene and that the *Natrans* gene had 100% identity with allele 2 (Suppl. Table 6). Accordingly, for the remaining 12 *P. larvae* isolates for which only ONT WGS data with the *Natrans* gene deletion was available, these isolates were also assigned *Natrans* allele 2 based on a percent allele similarity of 99.8% (Suppl. Table 6).

All ONT genomes were annotated using Prokka,^43^ and pangenome analysis was performed using the Roary pipeline.^37^ Maximum-likelihood phylogenetic analysis of the ONT genomes was performed based on the multiple sequence alignment of core genes using MAFFT^28^ and FastTree.^40^ Additionally, maximum-likelihood phylogenetic analysis was performed based on the presence or absence of accessory genes. Phylogenetic trees were visualized using iTOL.^32^

### Statistical analysis

Linear regression of LMC MIC values (µg/mL) on LMC Kirby–Bauer zone of inhibition (mm) was performed using STATA (Release 17; StataCorp).^45^

## Results

### Prevalence of OTC resistance among *P. larvae* isolates from Saskatchewan

**Figure 1.**
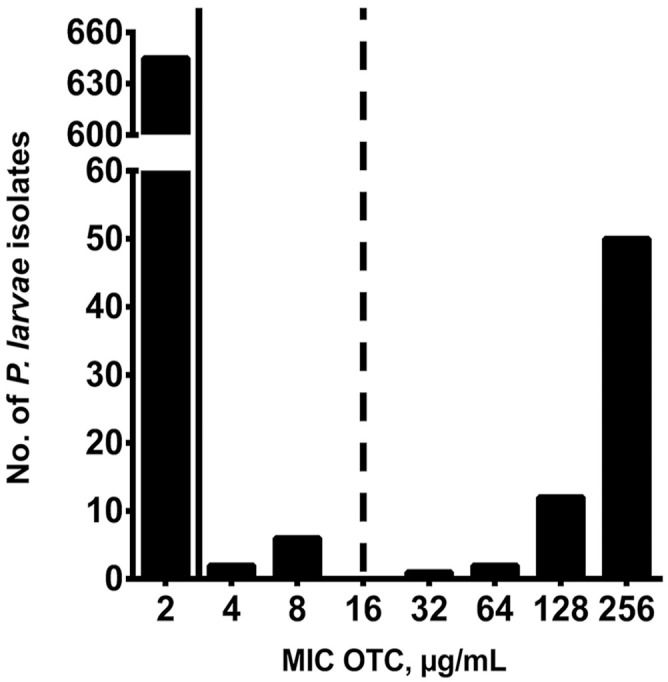
Oxytetracycline (OTC) susceptibility of *Paenibacillus larvae* isolates from Saskatchewan, Canada, from 52 commercial beekeeping operations in 2019 and 2020. Bars represent number of *P. larvae* isolates for each MIC value of OTC (µg/mL) for 718 *P. larvae* samples. Solid and dashed lines indicate the CLSI breakpoints for OTC sensitivity (≤2 µg/mL OTC) and resistance to OTC (≥16 µg/mL OTC), respectively.^15^

**Figure 2.**
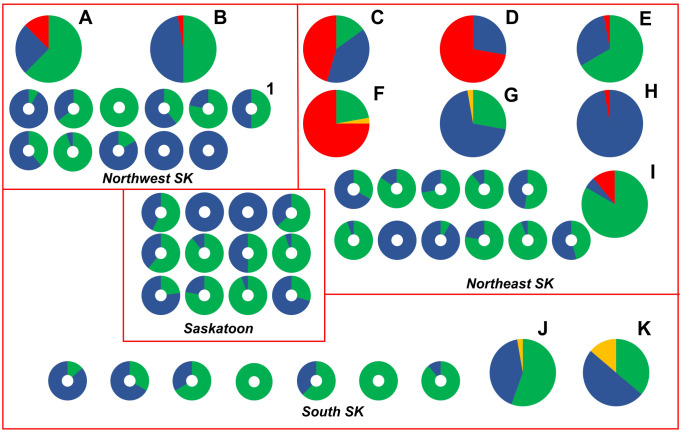
Spatial distribution and oxytetracycline (OTC) susceptibility of *Paenibacillus larvae* isolates from 1 of 52 Saskatchewan, Canada commercial beekeeping operations from 2019 and 2020. Each pie chart or donut chart represents the OTC susceptibility of *P. larvae* isolates cultured from the extracted honey in 1 of 4 (northeastern, northwestern, Saskatoon-area, southern) regions of Saskatchewan. Lettered pie charts represent beekeeping operations whose extracted honey samples contained resistant (red) and/or intermediate resistant (yellow) isolates of *P. larvae*. Donut-shaped charts represent commercial beekeepers whose *P. larvae* isolates were all susceptible to OTC (blue) or whose honey samples did not contain any detectable *P. larvae* spores (green). Donut chart “1” represents a beekeeper from whom an additional OTC- susceptible *P. larvae* isolate was characterized by whole-genome sequencing. The size of pie charts was increased relative to donut charts to enhance readability and does not reflect differences in sample size.

Of the 718 *P. larvae* samples tested, 645 (89.8%) were susceptible to OTC, 8 (1.1%) isolates had intermediate resistance to OTC, and 65 (9.0%) isolates were resistant to OTC, based on MIC determination using the agar plate and broth microdilution methods (Fig. 1). Most OTC-resistant isolates (60 of 65; 92.3%) were identified in 6 beekeeping operations (mean of 10.8 resistant isolates per beekeeping operation) located in the northeastern (NE) region of SK; the remaining 5 OTC-resistant isolates (7.7%) were identified in 2 beekeeping operations in the northwestern (NW) region of SK (Fig. 2). Accordingly, OTC-resistant *P. larvae* isolates were identified in 6 of 52 (11.5%) beekeeping operations surveyed. *P. larvae* isolates with intermediate resistance to OTC were identified in 4 of 52 (7.7%) beekeeping operations, with 2 affected operations in each of the NE and southern (S) regions of SK (Fig. 2).

For 64 of 73 (87.7%) isolates, there was agreement between the MIC and the Kirby–Bauer disk diffusion test for classification of the isolate as susceptible, intermediate resistant, or resistant to OTC. Among these 64 isolates, isolates with intermediate resistance to OTC had a mean zone of inhibition of 15.0 mm (SD: 0.5 mm) and OTC-resistant isolates had a mean zone of inhibition of 11.9 mm (SD: 1.5 mm).

For 8 of 73 (11.0%) isolates, there was a discrepancy in OTC susceptibility interpretation between the Kirby–Bauer disk diffusion and MIC results (Suppl. Table 3). Based on Kirby–Bauer testing, these 8 isolates were classified as OTC-susceptible; however, the MIC values for these isolates corresponded to either intermediate OTC-resistant (6 isolates) or OTC-resistant (2 isolates) classification. For all isolates, MIC values were used for final interpretation of OTC susceptibility.

### Susceptibility of Saskatchewan *P. larvae* isolates to TYL and LMC

**Figure 3.**
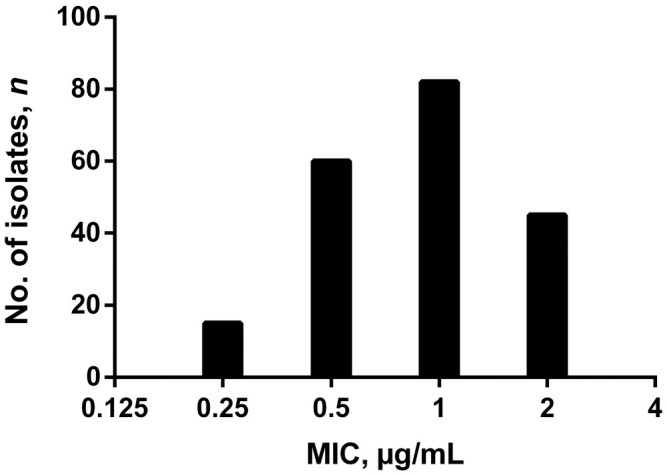
Lincomycin (LMC) susceptibility of 202 *Paenibacillus larvae* samples from 52 commercial beekeeping operations in Saskatchewan, Canada, from 2019 and 2020. Bars indicate number of *P. larvae* isolates with each MIC value of LMC (µg/mL).

**Figure 4.**
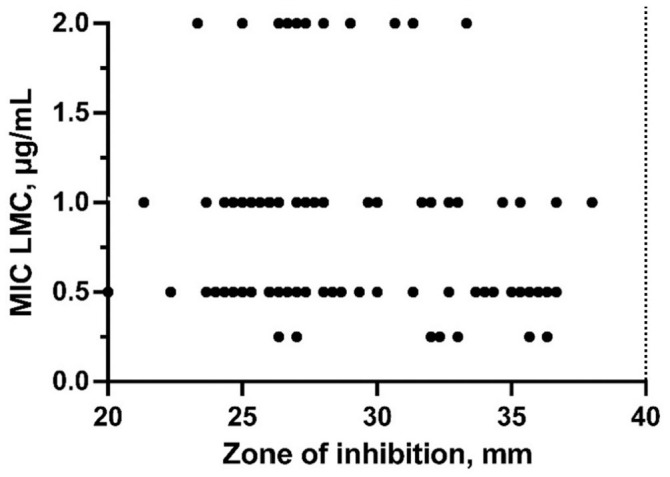
Relationship between MIC and Kirby–Bauer disk diffusion test results for lincomycin (LMC) susceptibility testing of 73 *Paenibacillus larvae* isolates (100 biological replicates) from Saskatchewan, Canada. Dots represent zone-of-inhibition diameter in mm for 4-µg LMC disks for *P. larvae* isolates with each MIC value of LMC (µg/mL). Dotted line indicates CLSI breakpoint zone diameter for susceptibility to LMC.^15^

All 718 *P. larvae* samples from the 52 beekeeping operations surveyed were susceptible to TYL, with MIC values ≤1 µg/mL, based on agar plate screening. Of the subset of 693 *P. larvae* samples tested for LMC sensitivity, 491 of 693 (70.8%) samples were susceptible to ≤0.125 µg/mL LMC, and the remaining 202 samples had MIC values of 0.25–2 µg/mL LMC (Fig. 3). Kirby–Bauer disk diffusion testing with 4-µg LMC disks indicated that *P. larvae* isolates with MIC values of 0.25–2 µg/mL LMC had a zone-of-inhibition diameter less than the susceptibility breakpoint of 40 mm (median: 28 mm, IQR: 26–32 mm; Fig. 4).^15^ Furthermore, a linear relationship between zone-of-inhibition diameter and MIC was not observed for 2-, 4-, 10-, nor 15-µg LMC disks (Fig. 4, Suppl. Fig. 1; LMC 2-µg *R*^2^ = 0.0452, LMC 4-µg *R*^2^ = 0.027, LMC 10-µg *R*^2^ = 0.02565, LMC 15-µg *R*^2^ = 0.0337).

### Quality control of antimicrobial susceptibility testing

The zones-of-inhibition diameter for *S. aureus* ATCC 25923 was within the accepted RI^16^ for OTC, and eMYPGP broth was able to support luxuriant growth of *S. aureus* ATCC 29213^15^ (Suppl. Table 3).

### Genetic determinants of AMR and pangenome analysis of *P. larvae* isolates in Saskatchewan

The *P. larvae*–specific resistance plasmid *pMA67* carrying the tetracycline-resistance gene *tet*(L)^36^ was identified in 9 of the 11 OTC-resistant isolates of *P. larvae* for which WGS was performed, representing 6 beekeeping operations from NE and NW regions of the province (Suppl. Table 6). WGS analysis of the remaining 2 OTC-resistant isolates of *P. larvae*, as well as the 3 OTC-susceptible isolates and the 3 intermediate OTC-resistant isolates, did not identify either *pMA67* or *tet*(L). The vancomycin-resistance genes (*vanF*, *vanH*, *vanW*, *vanT*, *vanY*) were also identified in all 17 *P. larvae* isolates sequenced. Two additional plasmids previously identified in *P. larvae* (unnamed plasmids 1 and 2)^7^ were present in 10 of 17 isolates (Suppl. Table 6).

Analysis of WGS assembly completeness revealed variable assembly quality among the *P. larvae* isolates sequenced. The ONT assemblies for isolates PL007–PL011 and PL013–PL018 (Suppl. Table 5) revealed 17–28 fragmented genes and 4–9 missing genes. In contrast, the assemblies of isolates PL001–PL006 had fewer fragmented genes (1–2) and no missing genes (Suppl. Table 5). For the isolates with both MiSeq and ONT WGS data (PL002–PL005), the assemblies were of comparable quality based on a similar number of fragmented and missing genes among the assemblies (Suppl. Table 5).

**Figure 5.**
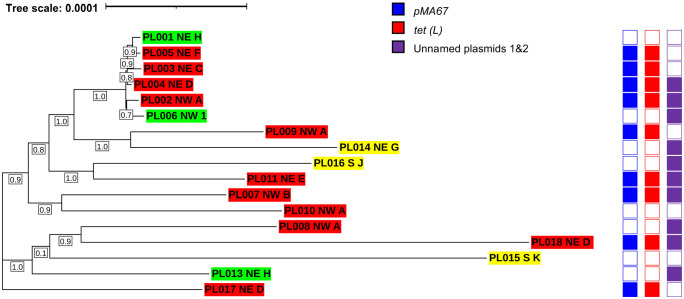
Maximum-likelihood phylogenetic tree of 17 *Paenibacillus larvae* isolates from 11 commercial beekeeping operations in Saskatchewan (SK), Canada, generated by FastTree^40^ based on multiple sequence alignment of the core genes. *P. larvae* isolates are indicated by isolate number followed by geographic region of origin (NE = northeastern SK; NW = northwestern SK; S = southern SK) and letter/number ID corresponding to beekeeping operation of origin. Oxytetracycline (OTC)-resistant isolates are highlighted in red; OTC-intermediate resistant isolates are highlighted in yellow; and OTC-susceptible isolates are highlighted in green. Scale bar corresponds to nucleotide substitutions per 100 bp. Bootstrap values are specified in boxes underneath the branches of the tree.

All 17 *P. larvae* isolates belonged to the ERIC I genotype and ST 15 (Suppl. Table 6). Pangenome analysis identified 14,593 genes, including 2,237 core genes, 6,357 accessory genes, and 5,999 cloud genes. Phylogenetic analysis based on multiple sequence alignment of core genes (Fig. 5), or presence and absence of accessory genes (Suppl. Fig. 2), revealed high genetic identity among the isolates with a maximum mean difference of 0.0003 nucleotide substitutions per site within the core genome. Phylogenetic clustering of the isolates varied between analysis of the core versus the accessory genome; however, in both analyses, there was no evidence of isolate grouping by geographic region, beekeeping operation, or phenotypic OTC-resistance profile.

## Discussion

OTC-resistant isolates of *P. larvae* have not been reported previously in SK, Canada, based on a search of the scientific literature indexed in Google Scholar, Web of Science, and Scopus. In our surveillance of 718 samples of *P. larvae* from 52 commercial beekeepers in SK from 2019 and 2020, we found that 11.5% of beekeeping operations harbored OTC-resistant isolates of *P. larvae*, with a spatial cluster of 60 OTC-resistant samples of *P. larvae* in 6 commercial beekeeping operations in the NE region of SK. Nevertheless, most (89.8%) *P. larvae* samples screened in our study were susceptible to OTC. Our study demonstrates the efficacy of pooled, extracted honey sampling as a surveillance tool for AMR in beekeeping operations. Although more comprehensive sampling of honey from SK beekeeping operations is necessary to estimate the true prevalence of OTC-resistant *P. larvae* in SK, our investigation identified high-risk geographic regions, including NE and NW SK, which should be a focus of future surveillance activity.

Similar to our study, past North American surveillance has identified tetracycline-resistant *P. larvae*,^31,46^ with identification of tetracycline-resistant *P. larvae* in up to 57% of diagnostic samples from Alberta, Canada in 2001,^21^ and sporadic detection of tetracycline-resistant *P. larvae* in Manitoba, Canada.^39^ Unfortunately, previous studies utilized variable experimental design, impeding meaningful comparisons among datasets, and arguing for a more standardized approach to the detection of OTC-resistant *P. larvae*, as presented here. For example, the sample type (adult bees, hive materials, larvae, honey), sample size, antibiotic tested (tetracycline vs. OTC), and sampling strategy (passive vs. active surveillance) vary among previous surveillance efforts for OTC-resistant *P. larvae* in North America. Considering the convenience of collection of pooled, extracted honey samples from commercial beekeeping operations, as well as the ability to monitor large geographic areas for OTC resistance without the need for individual hive inspections, we recommend that our methodology be applied to future surveillance efforts to enhance our understanding of the epidemiology of AMR in *P. larvae* in North America.

We observed disagreement in the determination of OTC sensitivity based on the broth microdilution test and the Kirby–Bauer disk diffusion test for 11% of *P. larvae* isolates for which results were available for both tests. This discrepancy could be explained by technical error in the Kirby–Bauer disk diffusion test, including uneven agar thickness resulting in variable antibiotic diffusion, or non-uniform distribution of bacterial inoculum. Similar to our results, other studies have found that bacterial isolates are more likely to be classified as antibiotic-resistant by MIC determination compared to Kirby–Bauer disk diffusion testing.^8^ In our study, MIC determination by the broth microdilution technique was the preferred test for screening of AMR in *P. larvae* because this test provides a clinically relevant outcome (antibiotic concentration in µg/mL) compared to the zone of inhibition (mm) provided by the Kirby–Bauer disk diffusion test. Moreover, as a secondary outcome to guide clinical therapy, the MBC can also be determined by the broth microdilution technique. Studies of AMR in *P. larvae* in North America^1,21,31,39,46^ have exclusively relied on the Kirby–Bauer disk diffusion test, highlighting the importance of MIC testing to enhance future *P. larvae* AMR surveillance.

Resistance plasmid *pMA67*, carrying the tetracycline-resistance gene *tet*(L), was found to explain phenotypic AMR in 9 of the 11 OTC-resistant isolates sequenced from SK, and has been reported in OTC-resistant *P. larvae* isolates from the United States and Canada.^24,34^ Unexpectedly, 2 of the OTC-resistant *P. larvae* isolates sequenced (PL008, PL010) from beekeeping operation A in NW SK, did not carry *pMA67* and *tet*(L), nor have other known genetic determinants of tetracycline resistance on WGS analysis. However, analysis of genome assembly completeness revealed a high number of fragmented and missing genes in the WGS assemblies for isolates PL008 and PL010, which may have resulted in the omission of *pMA67* and *tet*(L) as a result of technical artifact. Alternatively, the absence of known tetracycline-resistance genes in these isolates may be explained by a novel ARG in these isolates, or another non-genetic mechanism of AMR, such as biofilm formation. Moreover, we did not detect *tet*(L), nor other known tetracycline-resistance genes, in *P. larvae* isolates with intermediate resistance to OTC, either because of technical error in genome assembly or non-genomic or novel genetic determinants of resistance. Regardless of OTC-resistance phenotype, all *P. larvae* isolates sequenced carried the ARG *qacJ* gene, which confers resistance to quaternary ammonium compounds,^9^ and the vancomycin-resistance genes *vanF*, *vanH*, *vanW*, *vanT*, and *vanY*, which encode ligases that inactivate glycopeptide antibiotics.^22,38^ The identification of these additional ARGs in *P. larvae* was presumed to be an incidental finding, considering that vancomycin- and ammonia-based disinfectants are not approved for AFB metaphylaxis, and these genes have been reported in other *Paenibacillus* species.^38^

Genotyping, MLST, and phylogenetic analysis of a subset of *P. larvae* isolates in SK revealed a homogeneous population of *P. larvae*, without phylogenetic clustering of isolates by OTC-sensitivity phenotype, geographic area, or beekeeping operation of origin. All 17 *P. larvae* isolates belonged to the same genotype (ERIC I) and ST (ST15), which was previously identified in Canada, and is the most common genotype and ST in North America.^31,35^ Considering that OTC resistance in *P. larvae* appears to be mediated by horizontal gene transfer of *pMA67*, it is not surprising that there is no evidence of phylogenetic relatedness among *P. larvae* isolates with different OTC-sensitivity phenotypes. Similarly, no correlation was found between OTC-resistance phenotype and haplotype of *P. larvae* isolates from the United States and Canada.^21^ Furthermore, lack of phylogenetic discrimination by geographic region and the high degree of similarity of the core and accessory genome of *P. larvae* isolates suggests high connectivity among commercial beekeeping operations in SK with spread of *P. larvae* throughout the province, possibly through sale and exchange of bees and beekeeping equipment. Previous genetic characterization of outbreak-associated *P. larvae* isolates in Slovenia demonstrated both local and distant spread of *P. larvae* genetic clones,^49^ reinforcing the potential for widespread dissemination of OTC-resistant *P. larvae* clones in SK. Our ongoing and future surveillance will focus on molecular epidemiologic analysis of a larger sample size of *P. larvae* isolates from SK to understand the transmission of OTC resistance in SK beekeeping operations.

Resistance to TYL was not identified among the 718 *P. larvae* samples screened, and all 693 *P. larvae* samples tested for LMC resistance demonstrated MICs ≤2 µg/mL, suggesting that TYL and LMC may be appropriate alternatives to OTC for AFB metaphylaxis in operations in which OTC-resistant isolates have been identified. Similar to our results, other studies have found that OTC-resistant isolates retain their susceptibility to TYL and LMC. In contrast to our study, resistance to TYL and LMC was first reported in 2017 in 7.8% of 33 *P. larvae* field isolates from the United States screened for antimicrobial susceptibility using Kirby–Bauer disk diffusion testing.^29^ One limitation of our study is that the pooled honey samples used for *P. larvae* isolation and surveillance were sampled randomly and therefore may not fully characterize the *P. larvae* population circulating within SK.^47,48^

Investigation of the LMC susceptibility of our *P. larvae* isolates was hindered by lack of MIC breakpoint values for *P. larvae*. All *P. larvae* isolates tested for LMC susceptibility by the Kirby–Bauer disk diffusion assay had a zone-of-inhibition diameter less than the CLSI-defined 40-mm breakpoint for susceptibility to 4-µg LMC disks (median: 28, IQR: 26–32). This result suggests that these *P. larvae* isolates are not susceptible to LMC, but inhibition zone diameter breakpoint values for LMC-intermediate susceptibility and LMC resistance are not defined by CLSI, making interpretation of our Kirby–Bauer disk diffusion assay results difficult. Furthermore, we were not able to commercially source the CLSI-recommended 4-µg LMC disks, resulting in potential additional variability in our experimental 4-µg LMC disks, although the SD of 0.99 in our 4-µg LMC inhibition zone diameters for 100 *P. larvae* biological replicates was only slightly greater than the SD of commercially sourced 2-µg (SD = 0.48), 10-µg (SD = 0.62), and 15-µg (SD = 0.63) LMC disks. Accordingly, re-evaluation and establishment of LMC breakpoint values for the Kirby–Bauer disk diffusion assay and MIC testing of *P. larvae* is necessary for meaningful interpretation of LMC susceptibility testing of *P. larvae*.

To define susceptibility breakpoints for LMC, a linear relationship between MIC and zone-of-inhibition values for a population of *P. larvae* isolates is necessary. Unfortunately, we were unsuccessful in establishing a linear relationship between MIC and zone-of-inhibition diameter for *P. larvae* and LMC, possibly because of the narrow range of MIC values observed for the *P. larvae* isolates in our sample set. Future work to establish MIC and zone-of-inhibition breakpoints should utilize a population of *P. larvae* with a broader range of LMC-resistance phenotypes.^8,19,20^

## Supplemental Material

sj-pdf-1-vdi-10.1177_10406387231200178 – Supplemental material for Oxytetracycline-resistant Paenibacillus larvae identified in commercial beekeeping operations in Saskatchewan using pooled honey sampling Click here for additional data file. Supplemental material, sj-pdf-1-vdi-10.1177_10406387231200178 for Oxytetracycline-resistant Paenibacillus larvae identified in commercial beekeeping operations in Saskatchewan using pooled honey sampling by Oleksii Obshta, Michael W. Zabrodski, Tayab Soomro, Geoff Wilson, Fatima Masood, Jenna Thebeau, Marina C. B. Silva, Sarah Biganski, Ivanna V. Kozii, Roman V. Koziy, M. Fahim Raza, Midhun S. Jose, Elemir Simko and Sarah C. Wood in Journal of Veterinary Diagnostic Investigation
