# Supplementary material for: Oxytetracycline-resistant Paenibacillus larvae identified in commercial beekeeping operations in Saskatchewan using pooled honey sampling
Source: J Vet Diagn Invest. 2023 Sep 13;35(6):645–54. doi: 10.1177/10406387231200178 (PMC10621554; doi:10.1177/10406387231200178)
Supplement: sj-pdf-1-vdi-10.1177_10406387231200178 – Supplemental material for Oxytetracycline-resistant Paenibacillus larvae identified in commercial beekeeping operations in Saskatchewan using pooled honey sampling [file sj-pdf-1-vdi-10.1177_10406387231200178.pdf]

JVDI: Supplemental material

Obshta O, et al. Oxytetracycline-resistant *Paenibacillus larvae* identified in commercial beekeeping operations in Saskatchewan using pooled honey sampling

Supplemental Table 1. Flowchart of oxytetracycline hydrochloride (OTC) sensitivity testing of *Paenibacillus larvae*.

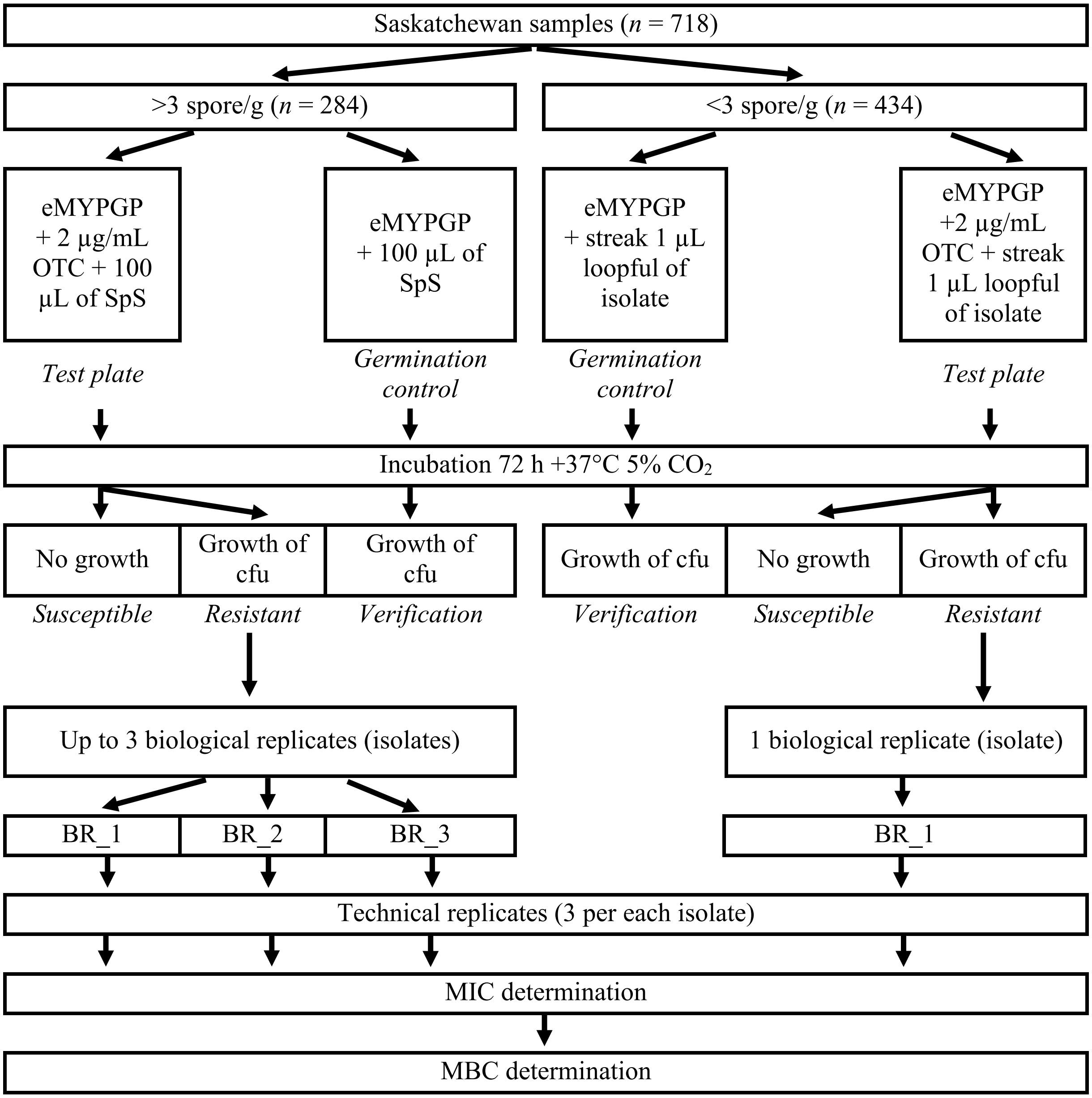

BR = biological replicate; eMYPGP = enhanced MYPGP medium; SpS = spore suspension.

Supplemental Table 2. Flowchart of lincomycin hydrochloride (LMC) and tylosin tartrate (TYL) sensitivity testing of *Paenibacillus larvae*.

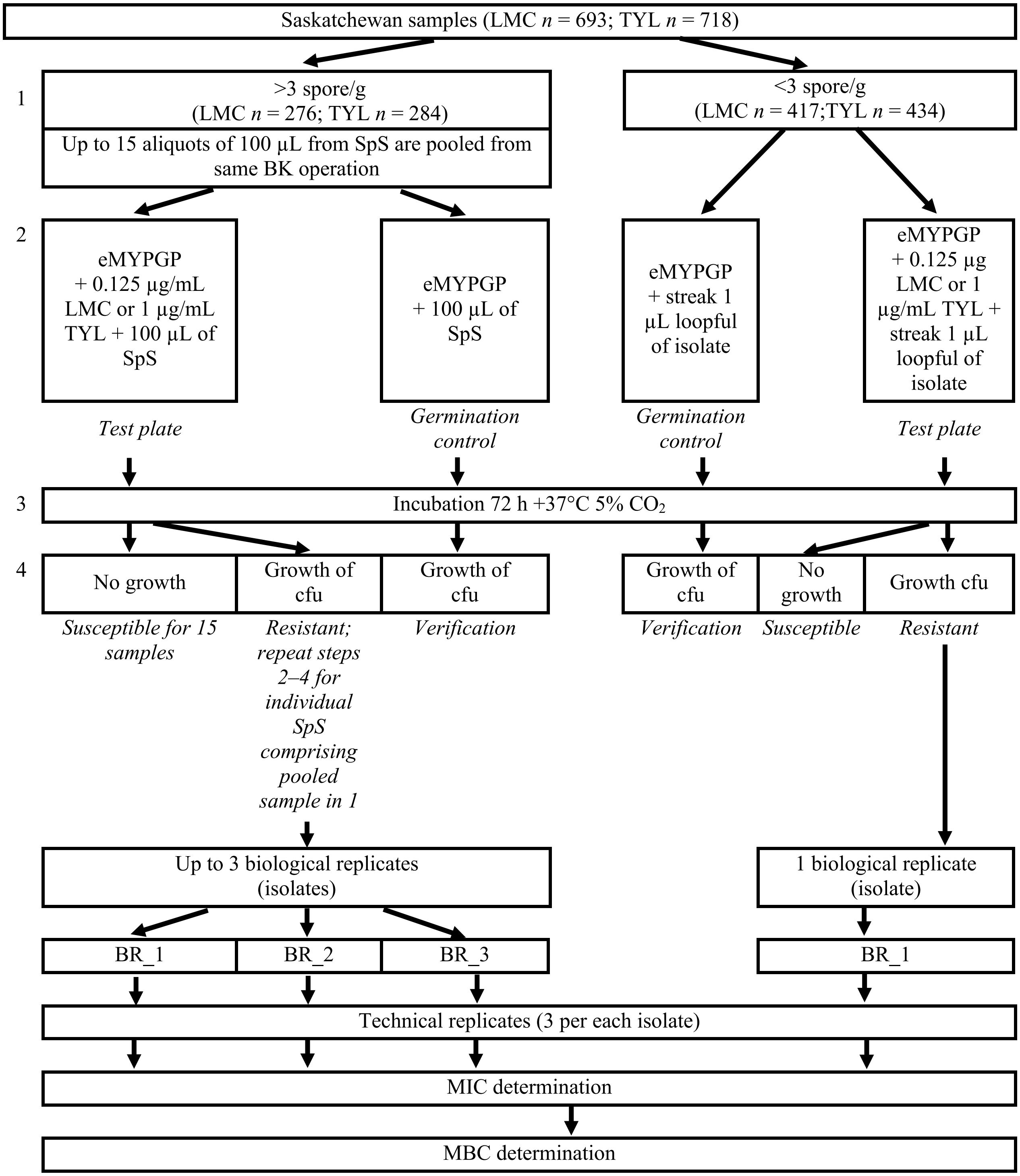

BR = biological replicate; eMYPGP = enhanced MYPGP medium; SpS = spore suspension.

Supplemental Table 3. Description of samples yielding isolates of *Paenibacillus larvae* with different oxytetracycline hydrochloride (OTC)-resistance phenotype in Saskatchewan, Canada commercial beekeeping operations.

No. of isolates	Year	Region of province	Spores/g of honey	MIC, µg/mL	MBC, µg/mL	Zone of inhibition, mm OTC 30-µg	Interpretation
Beekeeping operation C							
1	2019	NE	2.67	256	>256	12.33 ± 0.33	R
2	2019	NE	3.98	256	>256	11.83 ± 0.37	R
3	2019	NE	3.78	128	256	11.66 ± 2.05	R
4	2019	NE	1,583.33	256	>256	11.66 ± 0.47	R
5	2019	NE	1,475.95	256	>256	11.44 ± 1.42	R
6	2019	NE	1,359.87	128	>256	12.16 ± 0.37	R
7	2019	NE	2.50	256	256	14.33 ± 0.47	R
8	2019	NE	1,732.67	256	>256	12.55 ± 1.16	R
9	2019	NE	2,288.56	128	256	10.00 ± 0.00	R
10	2019	NE	1,416.67	256	256	12.11 ± 1.85	R
11	2019	NE	1,683.17	256	>256	13.00 ± 0.81	R
12	2019	NE	1,077.94	256	>256	12.33 ± 0.47	R
13	2019	NE	1,625.21	128	>256	12.11 ± 1.85	R
14	2020	NE	0.33	256	>256	12.00 ± 0.00	R
15	2020	NE	0.17	256	>256	9.0 ± 0.00	R
16	2020	NE	0.17	256	256	11.00 ± 0.00	R
Beekeeping operation G							
1	2019	NE	6.90	4	8	47.33 ± 0.94	I
Beekeeping operation F							
1	2019	NE	0.17	256	256	11.66 ± 0.47	R
2	2019	NE	10.50	256	>256	12.33 ± 0.47	R
3	2019	NE	8.29	256	>256	12.00 ± 0.57	R
4	2019	NE	0.16	256	256	14.33 ± 0.47	R
5	2019	NE	3.50	256	>256	8.66 ± 0.74	R
6	2019	NE	1.67	256	256	12.66 ± 0.47	R
7	2019	NE	8.58	256	>256	12.33 ± 0.47	R
8	2019	NE	2.31	256	256	13.66 ± 0.47	R
9	2019	NE	49.09	256	256	15.66 ± 3.77	R
10	2019	NE	3.12	256	>256	11.66 ± 0.47	R
11	2019	NE	1.15	256	256	12.66 ± 0.94	R
12	2019	NE	0.17	256	>256	11.00 ± 0.00	R
13	2019	NE	0.17	256	256	12.00 ± 0.00	R
14	2019	NE	0.67	256	256	10.66 ± 0.47	R

15	2019	NE	0.50	256	256	14.66 ± 0.47	R
16	2019	NE	0.33	256	256	10.00 ± 0.00	R
17	2020	NE	1.33	128	128	12.00 ± 0.00	R
18	2020	NE	5.00	256	>256	10.33 ± 0.47	R
19	2020	NE	0.83	256	256	10.33 ± 0.47	R
20	2020	NE	8.17	128	256	10.83 ± 0.68	R
21	2020	NE	32.83	256	>256	12.44 ± 0.83	R
22	2020	NE	6.00	256	>256	12.16 ± 1.06	R
23	2020	NE	16.83	256	>256	13.22 ± 0.62	R
24	2020	NE	0.17	8	32	10.33 ± 0.47	I
25	2020	NE	0.33	128	256	11.83 ± 0.68	R
26	2020	NE	1.00	64	128	12.33 ± 0.47	R
27	2020	NE	0.50	128	128	10.00 ± 0.00	R
28	2020	NE	18.33	256	256	13.33 ± 0.47	R
Beekeeping operation I							
1	2019	NE	0.67	256	256	12.00 ± 0.00	R
2	2019	NE	0.16	128	128	12.00 ± 0.00	R
Beekeeping operation E							
1	2019	NE	1.33	256	>256	43.00 ± 0.81	R
Beekeeping operation H							
1	2019	NE	4,233.33	<2	<2	44.66 ± 0.47	S
Beekeeping operation D							
1	2019	NE	115.51	256	>256	12.00 ± 0.00	R
2	2019	NE	325.04	256	>256	10.55 ± 1.34	R
3	2019	NE	160.13	256	>256	13.66 ± 0.47	R
4	2019	NE	173.27	256	>256	12.44 ± 0.83	R
5	2019	NE	6.47	256	256	13.33 ± 0.47	R
6	2019	NE	10.73	256	>256	13.22 ± 0.62	R
7	2019	NE	8.13	256	256	12.16 ± 1.21	R
8	2019	NE	5.72	256	>256	12.33 ± 1.37	R
9	2019	NE	3.40	128	256	11.33 ± 0.47	R
10	2019	NE	3.58	256	256	11.66 ± 0.47	R
11	2019	NE	9.59	256	256	12.16 ± 0.89	R
12	2019	NE	7.15	64	128	9.00 ± 0.81	R
13	2019	NE	5.00	256	256	12.33 ± 0.94	R
Beekeeping operation A							
1	2020	NW	12.83	128	256	8.66 ± 0.47	R
2	2020	NW	2.83	256	>256	10.66 ± 0.47	R
3	2020	NW	2.67	256	>256	10.83 ± 0.89	R
4	2020	NW	16.00	32	32	37.33 ± 0.94	R
Beekeeping operation B							
1	2019	NW	0.17	256	256	12.00 ± 0.00	R

Beekeeping operation K							
1	2020	So	1.00	8	32	43.33 ± 2.05	I
2	2020	So	0.33	4	16	42.66 ± 0.47	I
3	2020	So	1.00	8	16	38.66 ± 5.73	I
4	2020	So	0.50	8	8	38.66 ± 1.69	I
5	2020	So	0.67	8	256	11.00 ± 0.00	I
Beekeeping operation J							
1	2020	So	2.17	8	16	42.66 ± 2.49	I
Isolates used for quality control							
1	2022	*S. aureus* ATCC 25923	NA	<2	NA	25.66 ± 0.47	S
2	2022	*P. larvae* ATCC 9545	NA	<2	NA		S

I = intermediate; NA = not applicable; NE = northeast; NW = northwestern; R = resistant; S = susceptible; So = southern.

Supplemental Table 4. Origin, oxytetracycline susceptibility, multilocus sequence typing (MLST), and tetracycline-resistance genes identified in a subset of 17 isolates of *Paenibacillus larvae* from Saskatchewan, Canada, submitted for whole-genome sequencing.

Isolate	Region	Beekeeping operation ID	MIC OTC, µg/mL	Phenotype	MLST								*pMA67*	*tet*(L)	Unnamed plasmids 1&2
					glp F	*sig F*	*glp T*	*Natrans*	*rpo B*	*clp C*	*fts A*	ST			
PL001	NE	H	<2	S	3	1	1	2	4	2	4	15	–	–	
PL002	NW	A	256	R	3	1	1	2	4	2	4	15	+	+	+
PL003	NE	C	128	R	3	1	1	2*	4	2	4	15	+	+	
PL004	NE	D	256	R	3	1	1	2	4	2	4	15	+	+	+
PL005	NE	F	256	R	3	1	1	2	4	2	4	15	+	+	
PL006	NW	1	<2	S	3	1	1	2*	4	2	4	15	–	–	+
PL007	NW	B	256	R	3	1	1	2*	4	2	4	15	+	+	+
PL008	NW	A	32	R	3	1	1	2*	4	2	4	15	–	–	+
PL009	NW	A	128	R	3	1	1	2*	4	2	4	15	+	+	
PL010	NW	A	256	R	3	1	1	2*	4	2	4	15	–	–	
PL011	NE	I	256	R	3	1	1	2*	4	2	4	15	+	+	+
PL013	NE	H	<2	S	3	1	1	2*	4	2	4	15	–	–	+
PL014	NE	G	4	I	3	1	1	2*	4	2	4	15	–	–	+
PL015	So	K	8	I	3	1	1	2*	4	2	4	15	–	–	
PL016	So	J	8	I	3	1	1	2*	4	2	4	15	–	–	+
PL017	NE	D	256	R	3	1	1	2*	4	2	4	15	+	+	
PL018	NE	D	256	R	3	1	1	2*	4	2	4	15	+	+	+

I = intermediate-resistance; MIC OTC = minimum inhibitory concentration of oxytetracycline; NE = northeast; NW = northwestern; R = resistant; S = susceptible; So = southern; ST = sequence type.

* Indicates a 1 base pair deletion present in the *Natrans* allele.

Supplemental Table 5. Quality assessment of genome assemblies for 17 representative isolates of *Paenibacillus larvae* isolated from commercial beekeeping operations in Saskatchewan, Canada.

Isolate	GC content, %	N50 value	L50 value	Sequence size	Longest contig size	No. of contigs	Coverage
PL001	44.2	4508284	1	4550916	4508284	5	126
PL002	44.2	4508819	1	4513845	4508819	2	155
PL003	44.1	4096227	1	4665686	4096227	15	126
PL004	44.2	1084906	2	4626398	1784677	11	153
PL005	44.2	4507615	1	4548166	4507615	6	144
PL006	44.1	4452494	1	4452494	4452494	1	147
PL007	44.2	931030	2	4473125	1815214	10	83
PL008	44.2	1060716	2	4497827	1556428	12	165
PL009	44.1	1664342	2	4492714	1808943	14	164
PL010	44.1	2615064	1	4530330	2615064	12	105
PL011	44.2	9112551	2	4502106	1780475	10	164
PL013	44.1	2697032	1	4455651	2697032	10	201
PL014	44.2	861108	2	4514695	1816628	14	131
PL015	44.1	839279	2	4541423	1885599	20	115
PL016	44.1	3515110	1	4391837	3515110	3	106
PL017	44.2	937663	2	4511684	1813847	11	205
PL018	44.2	4464277	1	4469303	4464277	2	113
PL002IL	44.2	22278	46	4032756	98746	574	70
PL003IL	44.2	25232	45	3999080	98746	545	62
PL004IL	44.2	21866	53	4010485	79130	542	90
PL005IL	44.2	23279	46	4029447	93026	564	84

GC = percentage of cytosine and guanine in sequenced isolates; L50 = count of smallest number of contigs whose length sum makes up half of genome size; N50 = the sequence length of the shortest contig at 50% of the total assembly length; sequence size = total number of base pairs that constitute the genome of *P. larvae*. The region of Saskatchewan from which each isolate was collected is indicated. The sequence type of each isolate is defined by the alleles present at 7 reference gene loci (*glp F*, *sig F*, *glp T*, *Natrans*, *rpo B*, *clp C*, *fts A*). The presence (+) or absence (–) of the plasmid *pMA67*, unnamed plasmids 1&2, and the tetracycline-resistance gene *tet*(L) are indicated. All isolates carried additional antimicrobial resistance genes *qacJ*, *vanH*, *vanF*, *vanW*, *vanT*, *vanY*.

Supplemental Table 6. Results of BUSCO analysis for 17 representative isolates of *Paenibacillus larvae* sequenced using ONT sequencing technology. Each column in the table presents number and percentage of genes from the pool of BUSCO genes groups based on their presence and completeness in tested isolate.

Isolate	Complete BUSCOs (C), %	Complete and single-copy BUSCOs (S), %	Complete and duplicated BUSCOs (D), %	Fragmented BUSCOs (F), %	Missing BUSCOs (M), %	Total BUSCO groups searched, %
PL001	123	122	1	1	0	124
	98.4	97.6	0.8	1.6	0.00	100
PL002	122	121	1	2	0	124
	98.4	97.6	0.8	1.6	0.00	100
PL003	123	122	1	1	0	124
	99.2	98.4	0.8	0.8	0.00	100
PL004	123	122	1	1	0	124
	99.2	98.4	0.8	0.8	0.00	100
PL005	123	122	1	1	0	124
	99.2	98.4	0.8	0.8	0.00	100
PL006	123	122	1	1	0	124
	99.2	98.4	0.8	0.8	0.00	100
PL007	93	92	1	23	8	124
	75.0	74.2	0.8	18.5	6.5	100
PL008	93	92	1	24	7	124
	75.0	74.2	0.8	19.4	5.6	100
PL009	93	92	1	23	8	124
	75.0	74.2	0.8	18.5	6.5	100
PL010	95	94	1	25	4	124
	76.6	75.8	0.8	20.2	3.2	100
PL011	95	94	1	23	6	124
	76.6	75.8	0.8	18.5	4.9	100
PL013	95	95	0	23	6	124
	76.6	76.6	0.00	18.5	4.9	100
PL014	92	91	1	26	6	124
	74.2	73.4	0.8	21.0	4.8	100
PL015	100	99	1	18	6	124
	80.6	79.8	0.8	14.5	4.9	100
PL016	94	94	0	24	6	124
	75.8	75.8	0.00	19.4	4.8	100
PL017	103	102	1	17	4	124
	83.1	82.3	0.8	13.7	3.2	100
PL018	87	87	0	28	9	124
	70.2	70.2	0.00	22.6	7.2	100

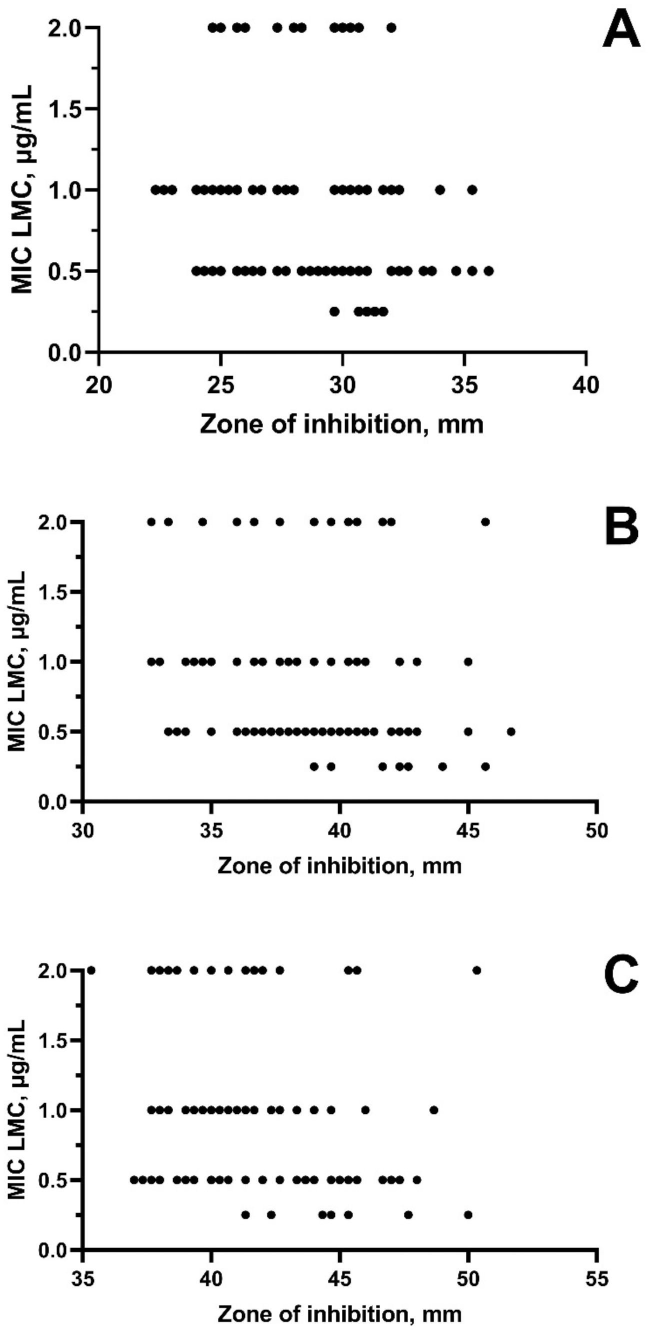

Supplemental Figure 1. Relationship between MIC and Kirby–Bauer disk diffusion test for lincomycin hydrochloride (LMC) susceptibility testing of 106 *Paenibacillus larvae* isolates from Saskatchewan, Canada. Dots represent zone of inhibition diameter in mm for 2-, 10-, 15-µg (**A, B,** and **C,** respectively) LMC disks for *P. larvae* isolates with each MIC value of LMC (µg/mL).

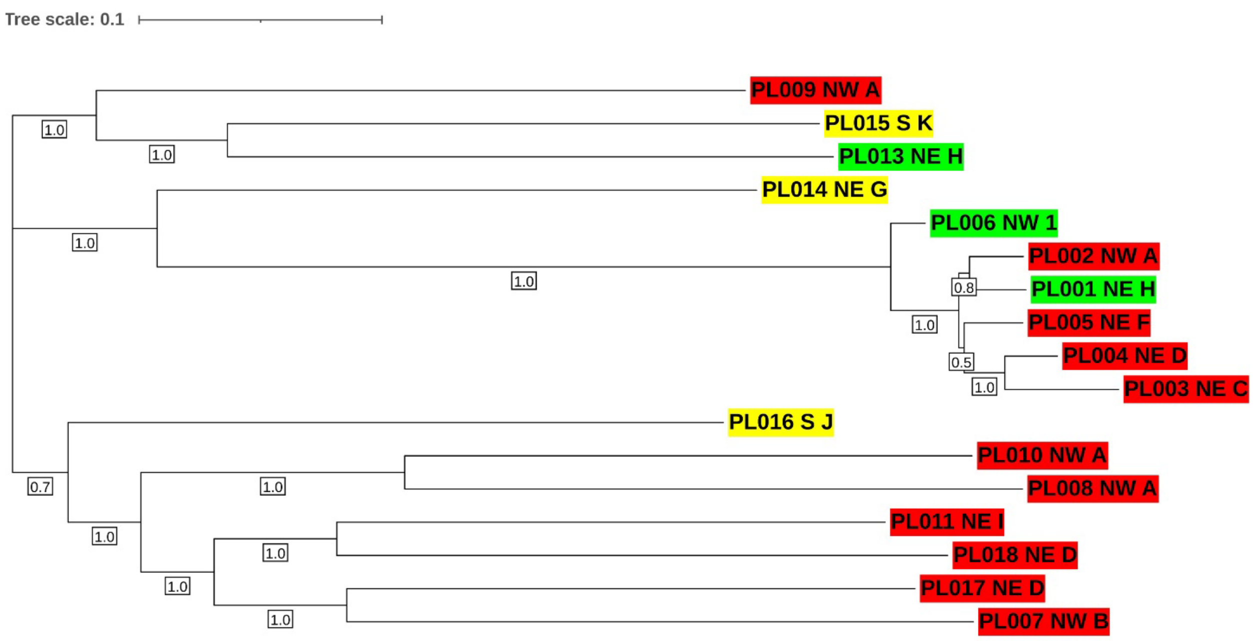

Supplemental Figure 2. Maximum-likelihood phylogenetic tree of 17 *Paenibacillus larvae* isolates from 11 commercial beekeeping operations in Saskatchewan (SK), Canada, generated by FastTree[37] based on binary presence/absence of accessory genes. *P. larvae* isolates are indicated by isolate number followed by geographic region of origin (NE = northeastern SK; NW = northwestern SK; S = southern SK) and a letter code for beekeeping operation. Oxytetracycline hydrochloride (OTC)-resistant isolates are highlighted in red; OTC-intermediate-resistant isolates are highlighted in yellow; and OTC-sensitive isolates are highlighted in green. Scale bar corresponds to nucleotide substitutions per 100 bp. Bootstrap values are specified in boxes underneath the branches of the tree.
